# Supplementary material for: High-Efficiency Ion Enrichment inside Ultra-Short Carbon Nanotubes
Source: Nanomaterials (Basel). 2022 Oct 9;12(19):3528. doi: 10.3390/nano12193528 (PMC9565519; doi:10.3390/nano12193528)
Supplement: Supplementary file 1 [file nanomaterials-12-03528-s001.zip › nanomaterials-1950406-supplementary.pdf]

Supplementary Materials: High-Efficiency Ion Enrichment Inside Ultra-Short Carbon Nanotubes

Yu Qiang [1,†], Xueliang Wang [2,3,†], Zhemian Ying [4,5], Yuying Zhou [4], Renduo Liu [4], Siyan Gao [1] and Long Yan [4,*]

[1] School of Physics and School of Material Science and Engineering, East China University of Science and Technology, Shanghai 200237, China
[2] Zhejiang Engineering Research Center for Tissue Repair Materials, Wenzhou Institute, University of Chinese Academy of Sciences, Wenzhou 325000, China
[3] School of physical sciences, University of Chinese Academy of Sciences, Beijing 100049, China
[4] Shanghai Institute of Applied Physics, Chinese Academy of Sciences, Shanghai 201800, China
[5] University of Chinese Academy of Sciences, Beijing 100049, China
* Correspondence: yanlong@sinap.ac.cn
† These authors contributed equally to this work.

Table S1. The mass of open nanotubes (M_{short}) and raw nanotubes (M_{raw}).

CNTs	M_{raw} (mg)	M_{short} (mg)	M_{short}: M_{raw}
MWCNTs (OD: ~6 nm)	1031	702	0.68
MWCNTs (OD: 20-50 nm)	1102	884	0.80

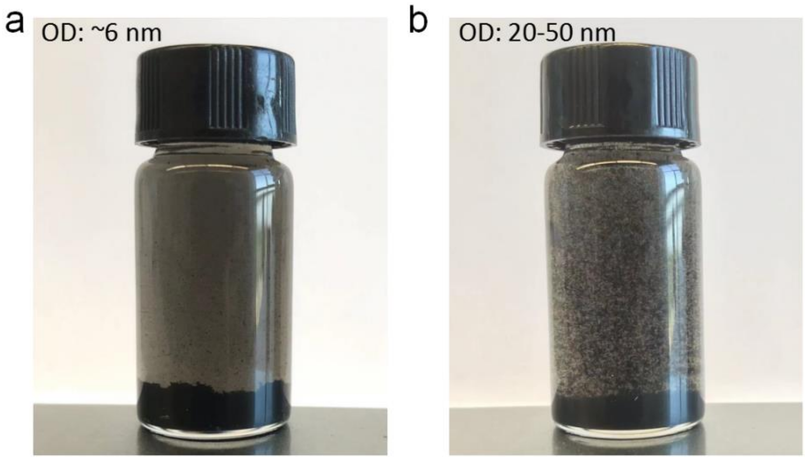

Figure S1. (**a**) A photograph of the suspension of the raw 6 nm-wide long multi-walled carbon nanotubes (MWCNTs). (**b**) A photograph of the suspension of the 20-50 nm-wide raw long MWCNTs. These two kinds of MWCNTs were dispersed in deionized water using ultrasonic treatment and then left standing for 1 h.

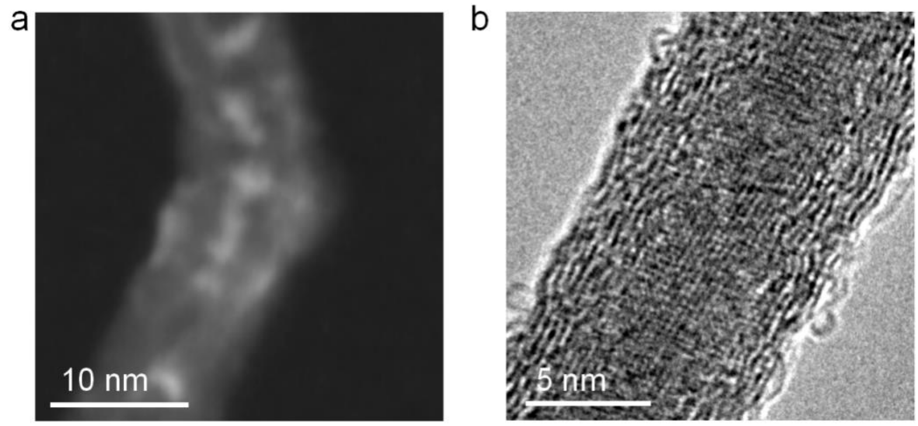

Figure S2. (**a**) A high-angle annular dark field scanning transmission electron microscopy (HAADF-STEM) image of a KCl aggregation blockage inside an MWCNT. (**b**) A high-resolution transmission electron microscopy (HRTEM) image of a crystalline KCl aggregation inside an MWCNT.

The K/C mass ratio of KCl aggregations inside CNTs

According to equation (5) and equation (6) in the manuscript, the mass of CNTs should be:

$$M = 0.25 \times 10^{-21} \times \pi l (0.5dN + 0.17N(N+1)) / 0.456 \qquad \text{(S1)}$$

where l is the length of the CNT, N is the total number of SWCNT layers and d is the inner diameter of the CNT.

If the CNT is filled with a dilute solution of the same concentration outside the nanotube, the mass of ion X should be:

$$m = 0.25 \times 10^{-24} \times \pi d^2 l \times C \times M_X \qquad \text{(S2)}$$

where the C is the concentration of the dilute solution and M_X is the relative atomic mass of element X.

Thus, the mass of ion / mass of carbon for a CNT filled with dilute solution is:

$$m / M = 4.56 \times 10^{-4} \times d^2 \times C \times M_X / (0.5dN + 0.17N(N+1)) \qquad \text{(S3)}$$

We assume a CNT has an inner diameter of 10 nm (d=10) and a wall number of 30 (N = 30). When the CNT is filled with KCl dilute solution, the value of C should be 0.14 mol/L and the M_X should be ~39.0 g/mol. According to equation S3, the assumed mass ratio of K/C, e.g., mass of K /mass of carbon (m/M) should be 0.00086.

The K/C mass ratios of the 16 aggregations inside 16 random ultra-short CNTs are displayed in Figure S3. The average K/C mass ratio of the 16 CNTs enriched K ions is 0.014, which is about 16.3 times as large as the assumed CNTs filled with KCl solution (0.14 mol/L). Considering the mass of carbon detected using EDS containing the amorphous carbon film of the TEM grid, we think the conclusion that KCl salt concentrations inside the short CNTs are 1–2 orders of magnitude higher than that of the solutions outside CNTs is reasonable.

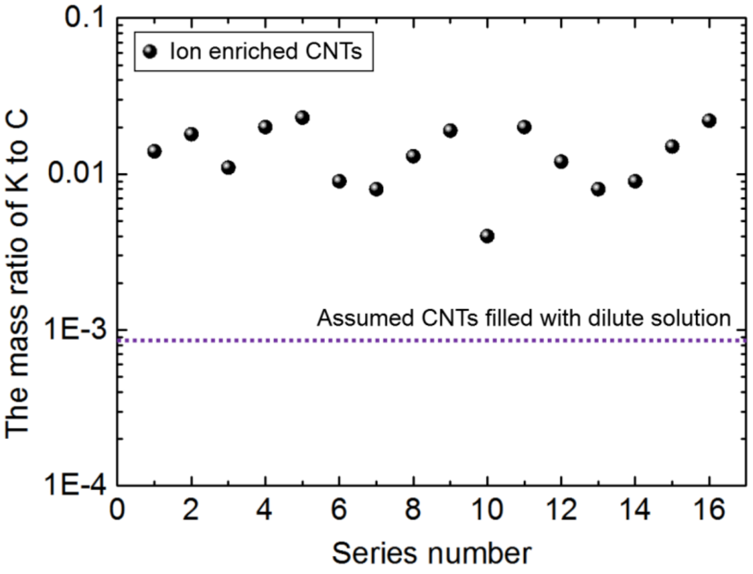

Figure S3. K/C mass ratios (black points) of the 16 aggregations inside 16 random ultra-short CNTs and the K/C mass ratios (dashed line) of assumed MWCNTs filled with dilute KCl solution (0.14 mol/L).

The filling capacity of the long CNTs

We characterized the long 20–50-nm-wide MWCNTs which were soaked in KCl solution (0.14 M) using TEM. The long MWCNTs were opened using acid pickling. It was easy to find salt aggregations near the mouth of the long MWCNTs (Figure 5a in manuscript). However, most of the mid-section part was empty due to the blockages preventing the movement of solutions inside the CNTs. Figure S4 displays the filling length to total CNT length ratio of 18 long 20-50 nm-wide MWCNTs, which were treated using acid pickling to open the CNT mouths. The average value was 0.029, much lower than that of the ultra-short CNTs (0.49).

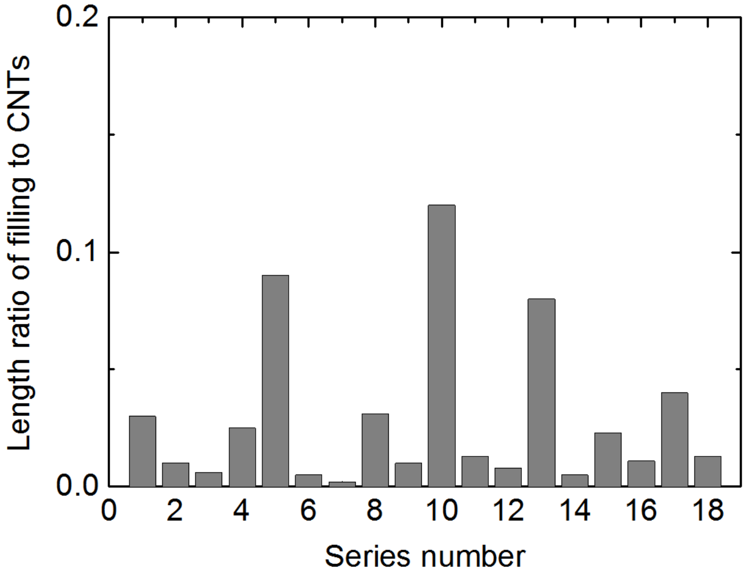

Figure S4. Filling length/total length ratios for 18 random long MWCNTs.

The Pb/C mass ratio of KCl aggregations inside CNTs

We also assume the CNT has an inner diameter of 10 nm (d = 10) and a wallnumber of 30 (N = 30). When the CNT is filled with $PbCl_2$ dilute solution, the value of C should be 0.14 mol/L and the M_X should be 207.0 g/mol. According to equation S3, the assumed mass ratio of Pb/C, e.g., mass of Pb /mass of carbon (m/M) should be 0.00429.

The Pb/C mass ratios of seven aggregations inside seven random ultra-short CNTs are displayed in Figure S5. The average of Pb/C mass ratios of the seven CNTs with enriched Pb ions was 0.09, which was about 21 times as large as the assumed CNTs filled with $PbCl_2$ solution. Considering the mass of carbon detected using EDS containing the amorphous carbon film of the TEM grid, these results prove that the ultra-short CNTs can enrich Pb ions.

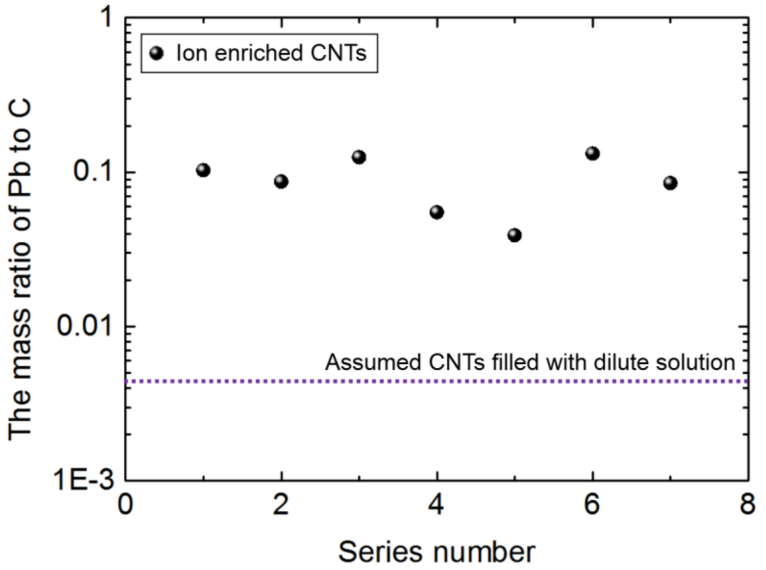

Figure S5. Pb/C mass ratios (black points) of the seven aggregations inside seven random ultra-short MWCNTs and the Pb/C mass ratios (dashed line) of assumed MWCNTs filled with dilute $PbCl_2$ solution (0.14 mol/L).

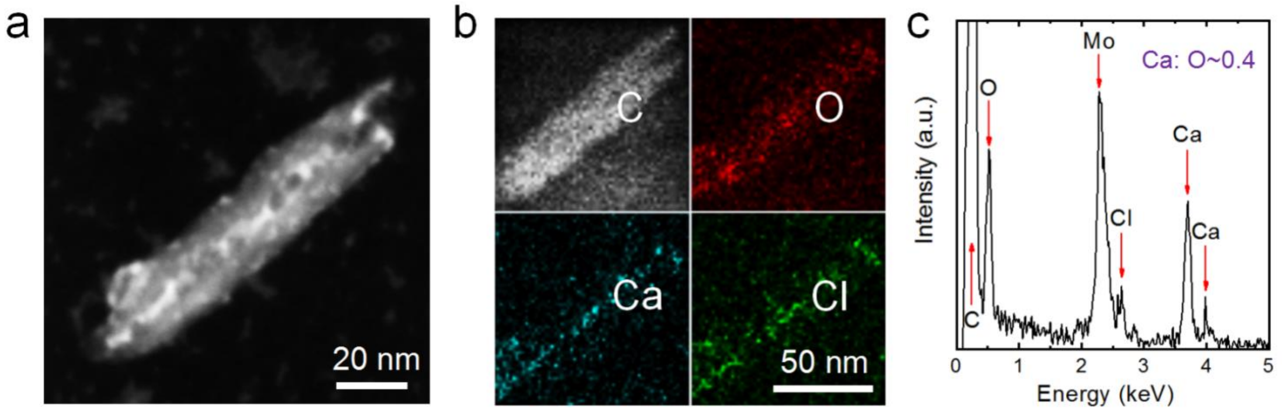

Figure S6. (**a**) An HAADF-STEM image of a $CaCl_2$ aggregation inside an ultra-short MWCNT. (**b**) The C, O, Ca and Cl element mappings of the region in the HAADF-STEM image in (**a**). (**c**) The energy-dispersive X-ray spectroscopy (EDS) result of the aggregation in (**a**). Here, the Mo signal in (**c**) comes from the Mo grids, which held the CNT.
